# Exploration of alternative splicing events in ten different grapevine cultivars

**DOI:** 10.1186/s12864-015-1922-5

**Published:** 2015-09-17

**Authors:** Emilio Potenza, Milvia Luisa Racchi, Lieven Sterck, Emanuela Coller, Elisa Asquini, Silvio C. E. Tosatto, Riccardo Velasco, Yves Van de Peer, Alessandro Cestaro

**Affiliations:** Fondazione Edmund Mach, Via E. Mach 1, 38010 S. Michele all’Adige, TN Italy; Department of Agri-Food Production and Environmental Sciences, Università degli Studi di Firenze, Firenze, 50121 Italy; Department of Plant Systems Biology, VIB, Ghent, Belgium; Department of Biomedical Sciences, Università degli Studi di Padova, Padova, 35131 Italy; Department of Plant Biotechnology and Bioinformatics, Ghent University, Ghent, Belgium; Department of Genetics, Genomics Research Institute, University of Pretoria, Pretoria, South Africa

**Keywords:** Alternative splicing, Vitis vinifera, RNA-seq, Stochastic noise

## Abstract

**Background:**

The complex dynamics of gene regulation in plants are still far from being fully understood. Among many factors involved, alternative splicing (AS) in particular is one of the least well documented. For many years, AS has been considered of less relevant in plants, especially when compared to animals, however, since the introduction of next generation sequencing techniques the number of plant genes believed to be alternatively spliced has increased exponentially.

**Results:**

Here, we performed a comprehensive high-throughput transcript sequencing of ten different grapevine cultivars, which resulted in the first high coverage atlas of the grape berry transcriptome. We also developed findAS, a software tool for the analysis of alternatively spliced junctions. We demonstrate that at least 44 % of multi-exonic genes undergo AS and a large number of low abundance splice variants is present within the 131.622 splice junctions we have annotated from Pinot noir.

**Conclusions:**

Our analysis shows that ~70 % of AS events have relatively low expression levels, furthermore alternative splice sites seem to be enriched near the constitutive ones in some extent showing the noise of the splicing mechanisms. However, AS seems to be extensively conserved among the 10 cultivars.

**Electronic supplementary material:**

The online version of this article (doi:10.1186/s12864-015-1922-5) contains supplementary material, which is available to authorized users.

## Background

The transcriptome is the collection of different RNA molecules, or transcripts, which are present in the cell at a given moment. For mRNA, a complementary RNA strand is first transcribed by RNA polymerase II and then spliced to produce mature mRNA by removing introns. The splicing process itself is performed by the spliceosome, a large RNA-protein complex that removes introns from pre-mRNA and ligates exons together [1]. Alternative splicing (AS) is a post-transcriptional process widespread in eukaryotic organisms that generates multiple distinctive transcripts from a single gene locus. It is generally accepted that AS events can be grouped into four main types: exon skipping (ES), intron retention (IR), alternative 5′ and 3′ (Alt-5′, Alt-3′) splice site [2]. Many studies have reported that the frequencies of these types can differ significantly between different kingdoms. For example, in several plants studies, IR has been confirmed as the prevalent type [3, 4]. However, the lack of extensive EST/cDNA collections, resulted in the fact that the real frequency of AS in plants has long been underestimated.

Nowadays, due to advances in high-throughput sequencing technology, detailed exploration of AS mechanisms has now become feasible [5, 6]. The most recent and accurate genome-wide investigation, carried out in *A. thaliana* using RNA-seq data, reported evidence of AS in over 61 % of intron-containing genes. RNA-seq analysis has become the standard method for genome-wide transcriptome analysis. It has the potential to overcome the limitation of previous technologies, mainly for its ability to detect novel mRNAs and produce millions of sequence reads [4, 7], providing the opportunity to investigate unknown AS aspects such as low-abundance events [8–10]. The unprecedented depth of sequence coverage has shown that even in humans a relevant part of the transcriptome is still not well characterized [11].

Like other fields in which NGS data is used, studying AS by means of RNA-seq has required the development of new computational tools. The majority of AS prediction software exploits algorithms derived from graph theory where genes are represented as DAGs (directed acyclic graph) [12]. The ways in which the DAG is walked through varies between methods, but in all cases the result is an estimate on the number of alternative transcripts. These estimations range from a minimum set that justifies the observed data (e.g. in CuffLinks ) to all possible paths, i.e all possible exons combinations [13, 14]. Such variability in the outcome is mainly due to the nature of RNA-seq generally used for expression analysis [13] such as the short read length.

Since its discovery, the relationship between organismal complexity and number of genes has greatly increased the interest in AS. Indeed, AS has been proposed to increase transcriptome and proteome complexity, for instance as a specific response to certain development stages or environmental stimuli. Moreover, AS can affect the activity, localization, stability and interaction capacity of a transcript [15–17]. Currently one of the major challenges is trying to understand which AS transcripts are really translated into proteins and thus contribute to an expanded proteome [18]. In humans, nearly all multi-exonic genes have an AS event, although most protein coding genes seem to have one major transcript expressed at a significantly higher level than others [11, 19, 20]. One explanation might be that most low-abundance alternative isoforms are likely to be nonfunctional and probably result from stochastic noise during the splicing process [19, 21]. It has also been proposed that intron length plays an important role for RNA degradation by means of non-sense mediated decay (NMD) mechanisms [22]. More recently, a noisy-splicing model was invoked to explain the roles of low-level AS transcripts and NMD in human cancer [18].

Grapevine (*Vitis* spp.) is one of the most ancient and economically important fruit crops worldwide (see FAO statistics at URL: http://faostat.fao.org/site/339/default.aspx). Many commercial products are directly derived from grapevine such as juice, fresh fruit, spirits, and of course wine. From the large family of the Vitaceae, almost all wine produced around the world is derived from *Vitis vinifera* [23]. Interest in understanding the development and maturation of grape berries is a consequence of the commercial relevance of the molecular features influencing berry and consequently wine quality.

Here, we performed a comparative genome-wide RNA-seq analysis of the berry transcriptome in ten different grapevine cultivars that were selected for their different metabolic profiles. This data provides the most comprehensive set of RNA-seq gene expression variants in grape to this moment, and is expected to facilitate detection of AS events at high resolution. We found evidence of AS in about 44 % of intron-containing genes with the majority of events showing a low-abundance coverage. We have identified many novel splice junctions that are extensively conserved between the ten analyzed cultivars. Rarely used splice sites seem to be enriched near constitutive splice sites, suggesting that a high number of nearly identical mRNAs is produced from a single gene locus.

## Methods

### cDNA library preparation for high-throughput sequencing

We selected ten *Vitis vinifera* cultivars with different metabolic profiles, of which seven black berry varieties (Pinot noir, Teroldego, Alicante Bouschet, Sangiovese, Moscato rosa, Lambrusco salamino, Cabernet franc) and three white berry varieties (Chardonnay, Ansonica and Kozma Poloskei Muskotaly). These varieties belong to Mattivi’s collection and has been selected to further understand their metabolic behavior previously described in his work [24]. To maintain a certain level of comparison we also collected the samples at the same development stage. Furthermore, all of them were of certified origin, checked, and named in agreement with existing literature and cultivated using a standardized system.

To facilitate the discrimination between differentially expressed AS events in *Vitis vinifera*, we decided to generate non-normalized libraries. Total mRNA was extracted from a pool of berries for each cultivar grown under normal conditions. All of these cultivars were sampled at technological maturity, defined as the content of soluble solids between 17 and 18° Bx. For each variety three independent samples were extracted and then pooled for RNA-seq analysis. Following the manufacturer instructions, ten cDNA libraries have been prepared with random primers using the TruSeq RNA Illumina kit. A global view of the grape berry transcriptome was obtained by sequencing the libraries using an Illumina GAIIx platform (85 bp paired-end reads).

### Read alignment to the *Vitis vinifera* reference genome

In total 206.394 million paired-end reads were generated (see Additional file 1: Table S1), with an average of 20 million per cultivar. Reads were filtered by dynamic end trimming with a Phred score of 30 as minimum quality and a minimum trimmed length of 50 bp. This step resulted in a strong reduction of the initial read numbers for each cultivar (from 7.42 to 17.38 %) as shown in Additional file 1: Table S1. Cleaned reads were aligned, using TopHat [25] software against the *Vitis vinifera* reference genome (PN40024 12X [26]). Software was used with standard parameters, except for the minimum intron length that was fixed at 25 nt [27]. For the gene prediction and annotation we used the V2.1 version (URL: http://genomes.cribi.unipd.it/DATA/V2/V2.1/) [28], up-to-date at the time of the analysis, for which is available also a functional annotation.

Several criteria were applied to evaluate alignment quality to be used for accurate discovery of novel splice junctions (SJs). First, a maximum of eight mismatches were allowed, a value that allows to cope with the unknown genetic variability between grape cultivars and reference genome. Second, only reads mapping uniquely on the genome were retained. Third, only spliced reads with a minimum length of eight nucleotides on the shortest end were kept. All thresholds were implemented to reduce the number of false positive AS [6]. Many splice junctions were identified from our alignments which were grouped in different categories, a splice junction falling inside the coordinates of an annotated gene is defined as “genic” otherwise “intergenic”, if the splice junction is not present in the corresponding gene prediction is defined as “novel” and eventually a splice junction is defined as “antisense” if the consensus sequence is located on the opposite strand to the one annotated on the gene prediction (Additional file 1: Figure S9). Furthermore, a splice junction is classified as coding sequence (CDS) if located inside the coding region, untranslated region (UTR) if located completely inside the untranslated region and UTR-CDS when one border is inside the UTR and the other inside the CDS.

In terms of relative amount of mapped reads, some cultivars show a very low performance, e.g. for the Kozma cultivar mapping efficiency was about 77 %. We further investigated this by performing an *ab initio* assembly of the entire sample using Trans-AbySS [29] (data not shown). This indicated that the many reads clustered together in regions annotated as ribosomal RNA. Furthermore, we have partially investigate other 3 cultivar (Cabernet, Chardonnay, Ansonica) again with and *ab initio* assembly (data not shown) but in those case the amount of ribosomal RNA is negligible. Going a bit deeper in the analysis and detecting hypothetical novel transcript, no reliable signal has been found to clearly discern the difference in mapping performance other then stringent mapping parameters and genetic variability between the cultivars.

### findAS: local AS identification

While available software is mainly designed for isoform reconstruction, we were solely interested in finding local alternative splicing events showing an alternative behavior on the splicing junction compared to the reference gene model [13, 14]. We developed a novel software tool to carry out detection of splice variants, called findAS (available on GitHub at https://github.com/aemilius1984/findAS). FindAS requires RNA-seq data aligned against a reference genome and gene coordinates. Aligned reads are grouped together and groups of overlapping alignments are defined as transcriptional units (TUs). Only TUs unambiguously associated with a single gene prediction are retained and compared against available exon coordinates, allowing to distinguish between different AS types. For this study we consider splice sites in which both the alternative as well as the constitutive form are supported by evidence of transcription. In case of IR we consider only the one supported by evidence of a complete intronic coverage but also evidence that the splice junction defining the intron truly exist in our data condition. We have also implemented special features to filter AS predictions by different sample coverage and to check the conservation of specific predictions among different samples. We only kept AS event predictions with evidence from three different cDNA libraries (e.g cultivars) to reduce the influence of sequencing and mapping errors for low-coverage events. A detailed description of the tool is available in the Additional file 1 and all predicted AS are provided as Additional file 2.

### Alternative Event Ratio

We defined a measure called Alternative Event Ratio (AER) to have an indication on the degree of expression for AS events. It is a simple measure reflecting the number of reads supporting the AS events relative to the number of reads in support of the canonical event. We calculated the AER for each AS type separately due to their different AS event nature. For intron retention, the AER was calculated as the median number of reads along the retained intron divided by the number of reads supporting the splice junction, as already described in Marquez et al. (IRR, intron retention ratio) [6]. For exon skipping, the ratio was calculated as the fraction of reads covering the alternative junction and the sum of reads covering the skipped constitutive junctions. Finally, alternative donor and alternative acceptor AER was simply the ratio between the alternative and constitutive splice sites.

### Functional annotation

We used gene ontology (GO) assignments to analyze the function of AS genes conserved in every cultivars. The reference GO annotation used is available at CRIBI web site (URL: http://genomes.cribi.unipd.it/DATA/V2/annotation/). To determine the over-representation of a certain term in each of our three gene subsets, a GO enrichment analysis has been performed using TopGO from Bioconductor (http://www.bioconductor.org/packages/release/bioc/html/topGO.html). The significance of occurrence for a certain GO term was determined using a Fisher’s exact test.

## Results

### Extensive coverage of the *Vitis vinifera* transcriptome

The differences in the number of reads mapped on the reference genome (PN40024) among cultivars broadly follows what is known about the genetic and metabolic relationships among different grape varieties. As expected, the highest level of mapped reads is obtained from Pinot noir for which 94 % of the cleaned reads could be aligned, while the lowest (77 %) is from the Kozma cultivar, a white variety originated from inter-crossing Hungarian grapevines. Ribosomal contamination has also to be taken into account for the low Kozma performance (see Material and Methods). All cultivars with a mapping efficiency above 90 % have a genetic link with Pinot (e.g. Teroldego [30]) or, if unrelated to Pinot (e.g. Lambrusco [31]), they share the same chemical profile for phenols (Lambrusco, Sangiovese and Moscato rosa [24]). The cultivars with few aligned reads belong to white varieties (Chardonnay and Kozma) or are related white varieties (Cabernet franc [23]). All alignments are of high quality, with perfect matches for 55.52 % of mapped reads (average for all cultivars) and the majority of the reads aligned in a unique place (average cultivar uniqueness: 92.32 %, Additional file 1: Table S2). Additionally, the alignments exhibit an extensive coverage for the whole grape genome (Additional file 1: Figure S1). These results confirm the great genetic variability among grape cultivars reinforcing the observation derived from transcriptome experiments in Shiraz and Corvina, where the amount of unmapped RNAseq data were 25 and 11 % respectively.

### Splice junction detection level in multiple cultivars

Despite high variability in the amount of genome covered by different cultivars, the relative number of new SJs identified is quite uniform. On average, for each cultivar we identified 120,208 SJs with a relatively small variation range (standard deviation 11 %) with the vast majority of SJs residing in previously annotated regions (97 %). Nonetheless, several new splice sites are identified, as new SJs account for 27 % (cultivar average) and the relative number of new positions is again similar for all cultivars (standard deviation 5 %). The discovery rate of new SJs does not seem to depend on the amount of raw data. Linear interpolation between number of reads and number of newly discovered SJs gives a Pearson correlation coefficient of 0.88 . With this correlation and a clear dependence on the number of mapped reads, we believe to have reached saturation for all mRNA produced by the berries at technological maturation. Moreover, the majority of SJs falls in coding exons (86.01 %), see Table 1 for detailed distribution of SJs within the gene model for each cultivar. Inspection of dinucleotides at the intron borders indicated an extensive usage of canonical plant splice site consensus sequences. We have identified 94.0 % GT-AG SJs, 2.1 % GC-AG and 0.7 % AT-AC, which is only slightly different from what has been observed in Arabidopsis [29].

**Table 1:** Splice junction discovery rate for each cultivar

Sample	SJs	Novel	Genic	(UTR; UTR-CDS; CDS)	Intergenic
Alicante Bouschet	114,393	25.46 %	97.42 %	10.39 %; 3.71 %; 85.90 %	2.58 %
Cabernet franc	113,553	25.35 %	97.32 %	10.12 %; 3.20 %; 86.67 %	2.68 %
Chardonnay	105,280	20.45 %	97.37 %	9.77 %; 2.55 %; 87.68 %	2.63 %
Ansonica	112,237	23.26 %	97.12 %	10.34 %; 2.78 %; 86.88 %	2.88 %
Kozma Palne Muskotaly	102,460	20.67 %	97.52 %	9.64 %; 2.57 %; 87.79 %	2.48 %
Lambrusco salamino	129,677	30.71 %	96.58 %	11.05 %; 3.88 %; 85.08 %	3.42 %
Moscato rosa	136,544	33.60 %	96.48 %	11.54 %; 4.06 %; 84.41 %	3.52 %
Pinot noir	131,622	31.22 %	96.75 %	11.14 %; 4.28 %; 84.58 %	3.25 %
Sangiovese	133,878	32.25 %	96.81 %	11.15 %; 4.12 %; 84.72 %	3.19 %
Teroldego	122,443	27.51 %	97.03 %	9.64 %; 2.57 %; 87.79 %	2.97 %
Average	120,209	27.05 %	97.04 %	10.55 %; 3.44 %; 86.01 %	2.96 %

In the 2^nd^ column is shown the total number of splice junctions (SJs) detected, in the 3^rd^ column the fraction of novel SJs and in the 4^th^ column SJs overlapping a gene prediction. In the columns 5 to 7 is shown the fraction of genic SJs annotated respectively inside UTR regions, UTR-CDS and CDS. The last column is shown the fraction of intragenic SJs

### Abundance of AS classes

**Fig. 1:**
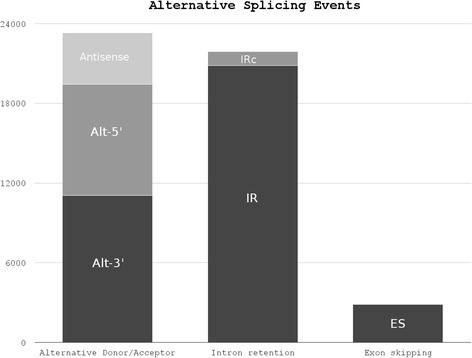
Alternative splicing types. The frequencies of the major categories of alternative splicing are shown in respect to the total amount of unique alternative splicing events identified within 10 *Vitis vinifera* cultivars

Among 25,341 gene models with more than one exon, we have information for 22,534 genes. Considering all data, we found 48,055 AS events with different conservation level among cultivars. 38,43 % (11,757) are shared by 3 cultivars and 21.69 % (6635) are AS conserved in each of our grapevine cultivars (Additional file 1: Figure S2). Overall, grouping together AS events detected in at least three cultivars, we found that 11.315 (44.65 %) of multi exonic genes are alternatively spliced. AS seems to occur mainly once or twice per gene (25.3 and 17.3 % of 22,534 respectively), but the extent of genes with one AS raises to 49.7 % considering those conserved among cultivars (Additional file 1: Figure S3). For each transcriptional unit we looked for AS falling into six main groups: exon skipping (ES), alternative 5′ donor site (Alt-5′), alternative 3′ acceptor site (Alt-3′), antisense splice junction (Antisense), intron retention (IR), and cryptic intron (IRc). As detailed in Table 2, the most common event is intron retention (43.35 %) and the least common exon skipping (5.91 %). These estimates agree with previous studies in other plants. Nevertheless, it is worth noting that exon junctions (either Alt-5′ or Alt-3′) account for 40.44 % of the total AS events (Fig. 1). The relative ratios among different AS events seems to be conserved in all cultivars (Table 2).

**Table 2:** Alternative splicing detection results. The frequency and overall raw count of the major categories of AS

Sample	Alt-3′	Alt-5′	Antisense	IR	IR cryptic	ES
Alicante Bouschet	5813	4223	1706	9216	451	1381
Cabernet franc	5501	4286	1629	9783	365	1503
Chardonnay	4599	3338	1317	7160	273	1046
Ansonica	5384	4069	1586	9612	399	1231
Kozma Palne Muskotaly	4304	3316	1204	7301	258	1143
Lambrusco salamino	7304	5447	2301	14,511	617	1719
Moscato rosa	7966	6010	2537	14,888	666	1890
Pinot noir	7542	5708	2419	14,609	611	1815
Sangiovese	7672	5808	2474	15,089	616	1903
Teroldego	6533	4939	2052	12,181	540	1575
Total Events	23.05 %	17.39 %	8.08 %	43.35 %	2.21 %	5.91 %
	11,077	8355	3883	20,834	1064	2842
	48.52 %			45.57 %		5.91 %

### Relatively low abundance of alternative events

**Fig. 2:**
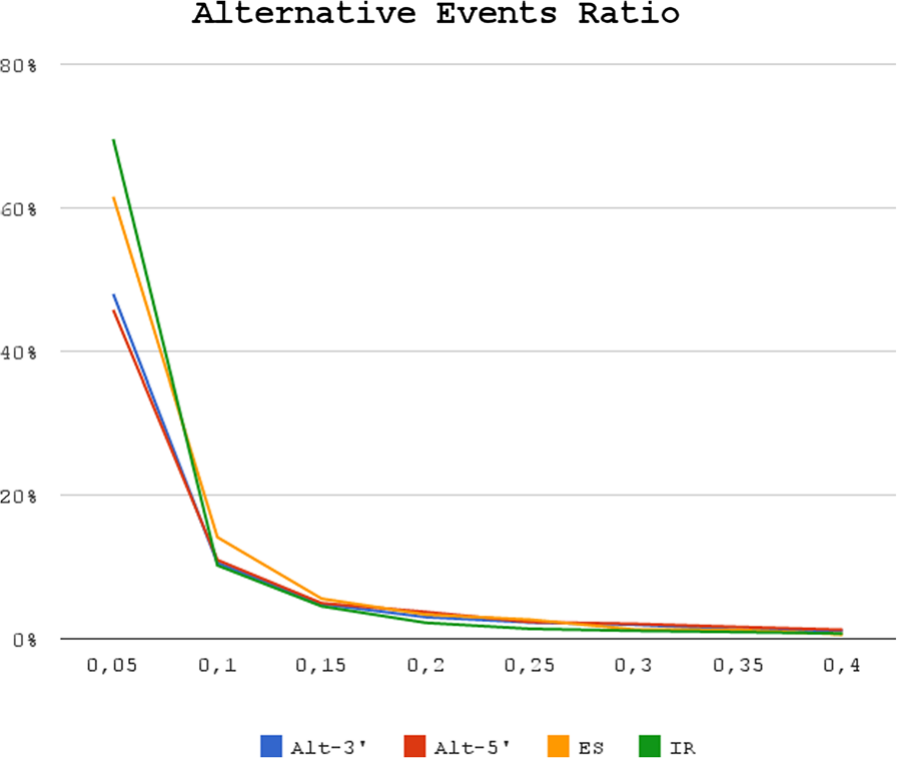
Alternative Events Ratio (AER). Relative coverage abundance between the putative alternative events conserved in all cultivars and related gene model. Each line represents the AS events fraction within an AER window of 0.05. The overall AS counts event conserved in all cultivars are: ES 453, IR 1962, IRc 142, Alt-3′ 2192, Alt-5′ 1595, Antisense 291

**Fig. 3:**
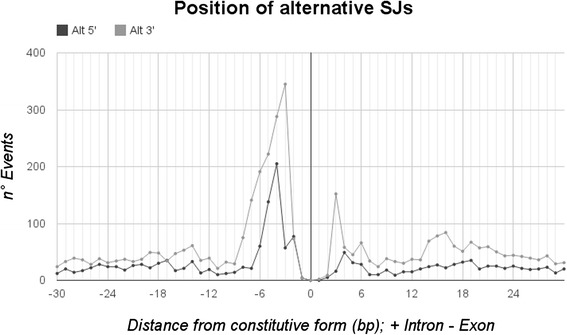
Distance of alternative SJs to the constitutive form. Alternative SJ positions (Alt-3′ and Alt-5′) to the relative SJ position annotated within the gene model

The expression level of potential transcript variations seems to be really low in our data. On average we found that ~70 % (of 43,108) AS are expressed less than 10 times with respect to their canonical form. This observation is confirmed for all kinds of AS, ranging from 56.7 % for Alt-5′ to 79.9 % of IR (Fig. 2). 9169 AS have a relatively high expression level compared to the canonical form (0.1 < =AER <1). Among these there are, potentially, peculiar isoforms of berry maturation. 1504 AS have an AER value equal or higher than one, a value indicating most likely errors in the gene structure rather than a high expression level. When looking at the distance of Alt-3′ and Alt-5′ junctions to constitutive exon borders, it is apparent that almost all events are located in a window less than 10 nucleotides from the canonical exon/intron border (see Fig. 3).

In order to evaluate if there is some evidence of periodicity and thus showing an over-representation for in-frame positions, we have divided AS events in two subcategories, AER <0.1 and 0.1 < =AER <1. We chose this threshold assuming that an AS with 0.1 < =AER <1 is more likely to play a functional role. Performing a binomial test with an expected random frequency of 33 % for the in-frame position, we found that in Alt-3′ and Alt-5′ with AER < 0.1 the positions not in frame is slightly more prevalent (31, 65 %, *P*-value = 0.03; 31.18 %, *P*-value = 0.038). On the other side, for Alt-3′ and Alt-5′ with 0.1 < =AER <1 the prevalence for the positions not in-frame is not relevant (34.26 %, *P*-value = 0.22; 33.42 %, *P*-value = 0.69).

### Functional annotation

We also analyzed gene function using gene ontology. In the subset of genes with AS conserved among all cultivars we counted 6635 genes only for 3820 of them had a functional annotation available. We perform the GO enrichment and we found an over representation of terms linked to “Intracellular Transport (GO:0046907) in the Biological Process ontology, “Translational factor activity” (GO:0008135) and “protein Ser/Thr phosphatase activity2 (GO:0004722) for “Molecular Function” ontology and “Nucleus” (GO:0005634) for “Cellular Component” ontology.

## Discussion

Alternative splicing is the most prominent mechanism to generate structural transcriptome complexity with two main different outcomes: proteome expansion and regulation of gene expression by premature stop codons. The latter results in down-regulation by nonsense-mediated decay or affects mRNA translation probability, localization and stability by means of UTR variability. Despite recent advances in sequencing technologies, plant transcriptome studies are still in their early stages. Even in well studied organisms, such as human, AS remains poorly understood. For example, recent evidence suggests that more than 90 % of human genes undergo AS [5, 32]. The functional role of such a high alternative transcript frequency is quite controversial and several studies suggest that the majority of these AS are simply due to noise introduced by the splicing machinery [11, 19, 22, 33], nevertheless it is important to note that AS play a important role for gene autoregulation by coupling AS with NMD [34].

We have decided to analyze AS without any attempt to reproduce neither entire transcripts nor the transcriptome. Avoiding the prediction of putative complete transcripts could appear a limitation, but this decision allows us to directly use our observations without any additional *a priori* assumptions [35]. Furthermore, one of our task was to investigate whenever an intraspecific alternative splicing events occur due to some sort of variability of individual splicing sites or maybe from the fine tuning of the spliceosome machinery, trying to investigate also low-abundance events. Processing this kind of information with a classical approach with Cufflinks [13], ASTALAVISTA [36] and the FPKM (Fragment Per Kilo base per Million) quantification of different isoforms can be tricky and lead to low quality results in the analysis of low abundance events, as recently was point out by Vitulo et al. [37].

Those are the main reason why we have also develop a new tool, findAS, that allowed us to fairly explore splicing junctions behavior without any constrain linked to the reconstruction of hypothetical full length transcripts.

The first basic outcome that it is important to note is that our results largely confirms the gene models used in the analysis. Aligned reads support the splicing pattern for 89.9 % of the predicted genes. Considering a certain level of stringency in our parameters we still have found AS evidence in 44.6 % of intron-containing genes, slightly more than the 30 % recently reported [37]. In term of relative expression for the observed alternative splicing junctions behavoiour we deduce that AS are quite frequent but poorly expressed, most of the time once or twice alternative spliced junctions per gene, moreover for those AS involving novel splice site we can also note that the novel SJ is often very close to the constitutive one.

Considering the different classes of AS our data are in agreement with what is already know in other plants [14] in which the IR is the most abundant class. Alternative splicing occurring in the opposite strand are present, on average, in 8 % of the genes, a number consistent with the frequency of Natural Antisense RNA (NAT) in A*rabidopsis thaliana* (9 % [38]) and rice (9.7 % [39]). Some studies had shown the role of alternative splicing for the regulation of: micro RNAs [40], antisense RNAs [41] and long non coding transcripts [42]. Antisense transcription is known to be widespread in many genomes; however, how much is functional is hotly debated. Specifically for our research we need further analysis to understand the role of the predicted antisense alternative splicing.

The number of predicted AS seems to correlate strongly with the total number of splicing reactions detected for each gene. The more splicing reactions a gene undergoes, either because it is highly transcribed or because it has many exons, the more AS were detected (Additional file 1: Figure S5). The AS number also seems in good correlation, even if not as strong as for the total number of splicing reactions, with the number of exons per gene and with the level of expression (Additional file 1: Figure S4 and S6).

Going deeply on AS behavior in different cultivars we were quite surprise to note that the conservation among them is definitely relevant, the average level of conservation that we have found is around 5 cultivars per alternative SJs events. Anyway, for what we could fairly demonstrated, the conservation does not seem to follow any known relation among grapevines.

We are not analyzing different conditions or tissues but just a pool of cultivars, but anyway the results we obtained are suggesting that in grapevine we have one main isoform per gene surrounded by many other relatively less abundant AS events, according with recent literature in grapevine [37]. Physico-chemical stochastic fluctuations of the cellular environment introduces small variability in the spliceosome efficiency and perhaps results in imperfect selection of splice sites that eventually produces many lowly expressed alternative transcripts, as is observed in our data [6, 13, 19, 33, 43, 44]. Besides the effect of stochastic noise on splice selection, recent studies are indicating that numerous low expressed AS isoforms will be a widespread regulatory mechanism functionally tuning the transcriptome [45]. Anyway, even if those low abundant events are produced randomly by a kind of stochastic noise or by a fine tuning in the splicing machinery, potential AS transcripts are definitively under natural selection in an evolutionary context.

## Conclusion

In conclusion, it should be considered that small variability in the splicing mechanism could play an important role for the plant adaptation. Considering the fact that even small changes in the transcript can possibly degenerate with the inclusion of stop codon and be part of an autoregolative pathway through the NMD. The effect of this selection could probably explain why many short AS have low expression yet are conserved among the 10 different cultivars. One may argue that, along with function, gene sequences might be selected to be able to tolerate a certain number and kind of AS events. In this light, both the number and impact of alternative transcripts should be reconsidered. While some data is already supporting this possibility for human transcripts [46], additional studies will be required in plants to clarify the importance of AS. We believe our work is an another important step towards the elucidation of SJs behavior and alternative splicing in plants.

## Availability of supporting data

The sequence data sets supporting the results of this article are available in the European Nucleotide Archive (http://www.ebi.ac.uk/ena) under the unique persistent identifier PRJEB9534.

The software developed for the analysis is available under General Public License Version 3 (GNU) in the GitHub repository (https://github.com/aemilius1984/findAS).

Other data supporting the results of this article are included within the article (and its additional files).

## Additional files

Additional file 1: **Supplementary material.** (DOCX 451 kb)

Additional file 2: **Table with all AS predicted by FindAS software.** First row (beginning with '#') hold the column labels. (ZIP 1893 kb)

## Abbreviations

AS: Alternative splicing
ES: Exon skipping
IR: Intron retention
Alt-5′: Alternative 5′ acceptor
Alt-3′: Alternative 3′ donor
DAG: Directed Acyclic Graph
NMD: Non sense Mediate Decay
CDS: Coding sequence
UTR: UnTraslated Region
AER: Alternative Event Ration
GO: Gene ontology
NAT: Natural AnTisense RNA
