# Supplementary material for: Exploration of alternative splicing events in ten different grapevine cultivars
Source: BMC Genomics. 2015 Sep 17;16(1):706. doi: 10.1186/s12864-015-1922-5 (PMC4574008; doi:10.1186/s12864-015-1922-5)
Supplement: Supplementary file 1 — Supplementary material. (DOCX 451 kb) [file 12864_2015_1922_MOESM1_ESM.docx]

**Additional file 1: Supplementary material**

**Supplementary Methods**

Below is an overview of findAS algorithm (see Supplementary Figure 7):

*Information source.* The starting point of our pipeline is a BAM file with fully cleaned alignments and a GFF file containing available gene predictions. These can be generated with any mapping software that fulfills two mandatory requirements: reads aligned over splice junctions (novel or previously annotated) and results should be in BAM format. Moreover, the software is capable of using reads from techniques other than Illumina and even mixed data.

*Primary clustering.* Alignments are first clustered according to their genomic position. Reads are grouped together if they overlap by at least one nucleotide. A transcriptional unit (TU) is defined as a group of continuous alignments spanning more than 50 nt.

*Chimera search.* After clustering, the resulting loci are uniquely linked to a gene model described in the GFF file. The software only assigns unambiguous transcriptional units, i.e. a TU should identify a single gene. This is not always possible, as a TU sometimes overlaps with more than one gene model, which we will call a chimeric locus. These could potentially represent a trans-splicing event, but this phenomenon is very hard to define. We simply prefer to mark the TU for further analysis and to remove it from the following AS detection. In case a TU contains more than one gene model, iterative clustering is performed to remove all ambiguous alignments until the chimera is no longer detected.

*AS detection.* Each group of aligned reads is compared against the available exon coordinates, allowing the distinction between different types of AS. When a difference between reads and exon position is detected, an AS instance is recorded and classified into 6 categories: exon skipping (ES), alternative 5' donor site (Alt-5’), alternative 3' acceptor site (Alt-3’), antisense splice junction (Antisense), intron retention (IR), cryptic intron (IRc). A junction is called “antisense” when the consensus sequence matches the complementary gene model strand. We define an intron as cryptic when it is found completely inside an annotated exon.

*Evidence check.* In order to predict an AS event, the alternative as well as the constitutive form must be both supported by transcript evidence. We decided to apply this criterion to avoid detection of AS without any direct evidence for the correctness of the gene model itself. Every AS event must therefore be covered by a minimum level of evidence. A coverage filter is applied to both the constitutive and alternative form, retaining only events with a cumulative coverage of at least 3 reads in at least 3 different cultivars. Moreover, each AS event type has its own cut-off. ES has a minimum required read coverage for both the consecutive exons and the SJs of the skipped exon. IR is detected if sufficient read depth is available inside the intron and for the SJs that defines the gene model intronic border. Alt-5’, Alt-3’ and antisense are detected only if a minimum read depth is reached on both the alternative SJ and constitutive forms (see Supplementary Figure 8).

In conclusion, even in case a gene is not completely covered by evidence, i.e alignments, due to expression related behavior or gene model misconception, findAS is able to detect AS events that are supported by evidence of at least 2 putative isoforms by checking the agreement between the alternative events and the gene model in the immediate surroundings of the putative AS.

**Table S1.** Sequencing and filtering results for different cultivars. Number of paired reads initially generated by the Illumina sequencing platform, the read pairs that passed our filters and the number of mapped reads (cleaned reads are available at the European Nucleotide Archive under the study ID PRJEB9534).

| **Sample** | **# Raw** | **# Cleaned (%Diff)** | | **# Mapped (%Clean)** | |
| --- | --- | --- | --- | --- | --- |
| **Alicante Bouschet** | 16,148,936 | 14,950,506 | -7.42% | 13,124,930 | 87.79% |
| **Cabernet franc** | 19,416,930 | 17,597,497 | -9.37% | 13,869,896 | 78.82% |
| **Chardonnay** | 17,816,446 | 15,350,304 | -13.84% | 12,559,770 | 81.82% |
| **Ansonica** | 23,344,136 | 20,008,317 | -14.29% | 16,615,248 | 83.04% |
| **Kozma Palne Muskotaly** | 21,237,030 | 17,546,066 | -17.38% | 13,641,795 | 77.75% |
| **Lambrusco salamino** | 22,357,539 | 20,214,750 | -9.58% | 18,449,293 | 91.27% |
| **Moscato rosa** | 24,864,531 | 22,585,359 | -9.17% | 20,567,709 | 91.07% |
| **Pinot noir** | 22,443,561 | 20,329,915 | -9.42% | 19,214,467 | 94.51% |
| **Sangiovese** | 22,181,869 | 20,099,091 | -9.39% | 18,821,173 | 93.64% |
| **Teroldego** | 16,583,320 | 15,014,666 | -9.46% | 13,676,524 | 91.09% |
| *TOTAL* | 206,394,298 | 183,696,471 | -11.00% | 160,540,805 | 87.39% |

**Table S2.** Mapping results for different cultivars. Total number of alignments, those obtained as unique match and the amount of alignment obtained without mismatch or with maximum one.

| **Sample** | **# Alignments** | **# Unique (%Align)** | | **# Perfect (%Align)** | | **# 1 Mismatch (%Align)** | |
| --- | --- | --- | --- | --- | --- | --- | --- |
| **Alicante Bouschet** | 27,492,097 | 25,396,830 | 92.38% | 13,910,730 | 50.60% | 6,123,044 | 22.27% |
| **Cabernet franc** | 29,029,405 | 26,887,112 | 92.62% | 12,991,100 | 44.75% | 5,463,438 | 18.82% |
| **Chardonnay** | 25,940,046 | 24,193,482 | 93.27% | 12,478,027 | 48.10% | 4,037,160 | 15.56% |
| **Ansonica** | 34,416,705 | 31,926,739 | 92.77% | 16,031,796 | 46.58% | 5,417,173 | 15.74% |
| **Kozma Palne Muskotaly** | 28,211,356 | 26,133,802 | 92.64% | 12,056,409 | 42.74% | 3,735,404 | 13.24% |
| **Lambrusco salamino** | 38,592,640 | 35,716,700 | 92.55% | 23,364,691 | 60.54% | 7,642,474 | 19.80% |
| **Moscato rosa** | 43,449,616 | 39,685,763 | 91.34% | 25,809,439 | 59.40% | 8,470,563 | 19.50% |
| **Pinot noir** | 40,408,356 | 37,195,504 | 92.05% | 27,028,102 | 66.89% | 7,245,484 | 17.93% |
| **Sangiovese** | 39,453,439 | 36,485,775 | 92.48% | 24,821,228 | 62.91% | 7,811,662 | 19.80% |
| **Teroldego** | 28,829,915 | 26,404,196 | 91.59% | 17,961,004 | 62.30% | 5,154,332 | 17.88% |
| *TOTAL* | 335,823,575 | 310,025,903 | 92.32% | 186,452,526 | 55.52% | 61,100,734 | 18.19% |


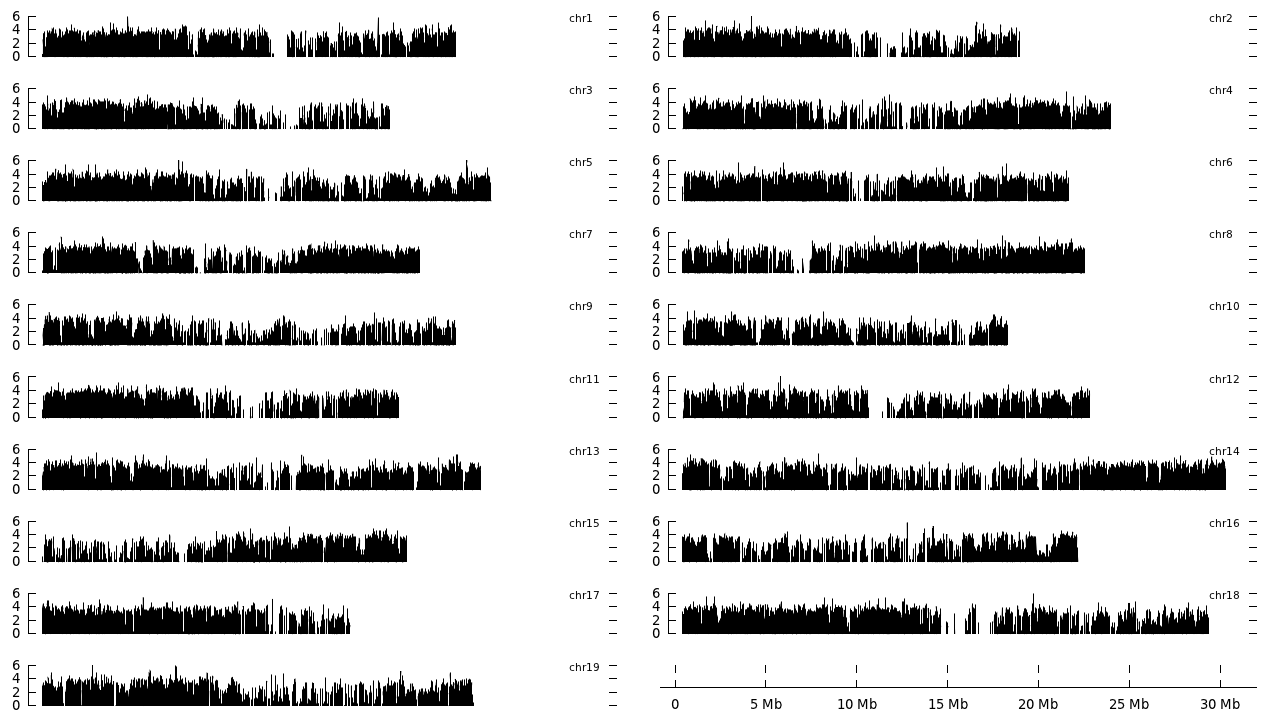


**Figure S1.** Log2 scale of read coverage in windows of 1 kb per chromosome. The distribution of RNA-seq coverage as sum of each cDNA library is shown along PN40024 reference genome chromosomes. A vertical black line refers to a log2 of the number of reads in a 1 kb window plotted against the chromosome coordinates.


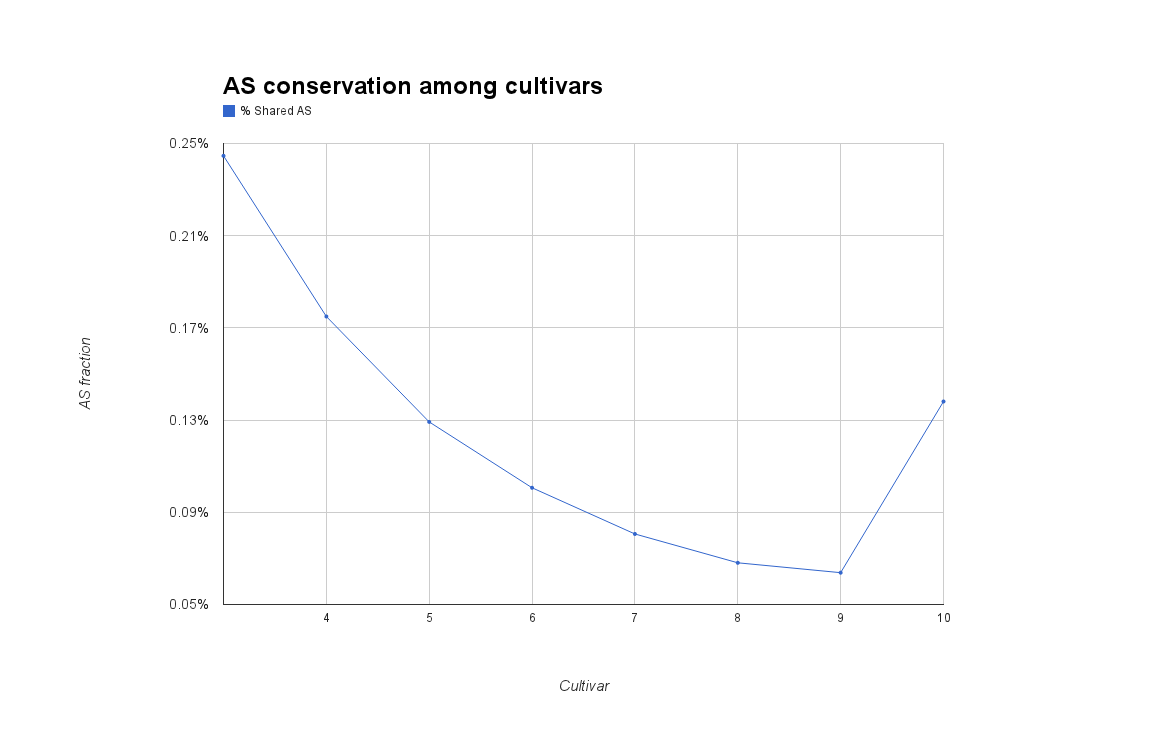


**Figure S2.** Relative abundance of AS events shared between cultivars. The number of shared AS events among different cultivars at various levels range from 11,757 in 3 cultivars to 3,066 events common to 9 cultivars. Notably, the number of alternative splices conserved among all cultivars 6,635 (13.81% of total predictions) is still relevant (15-16 % of the whole gene set).


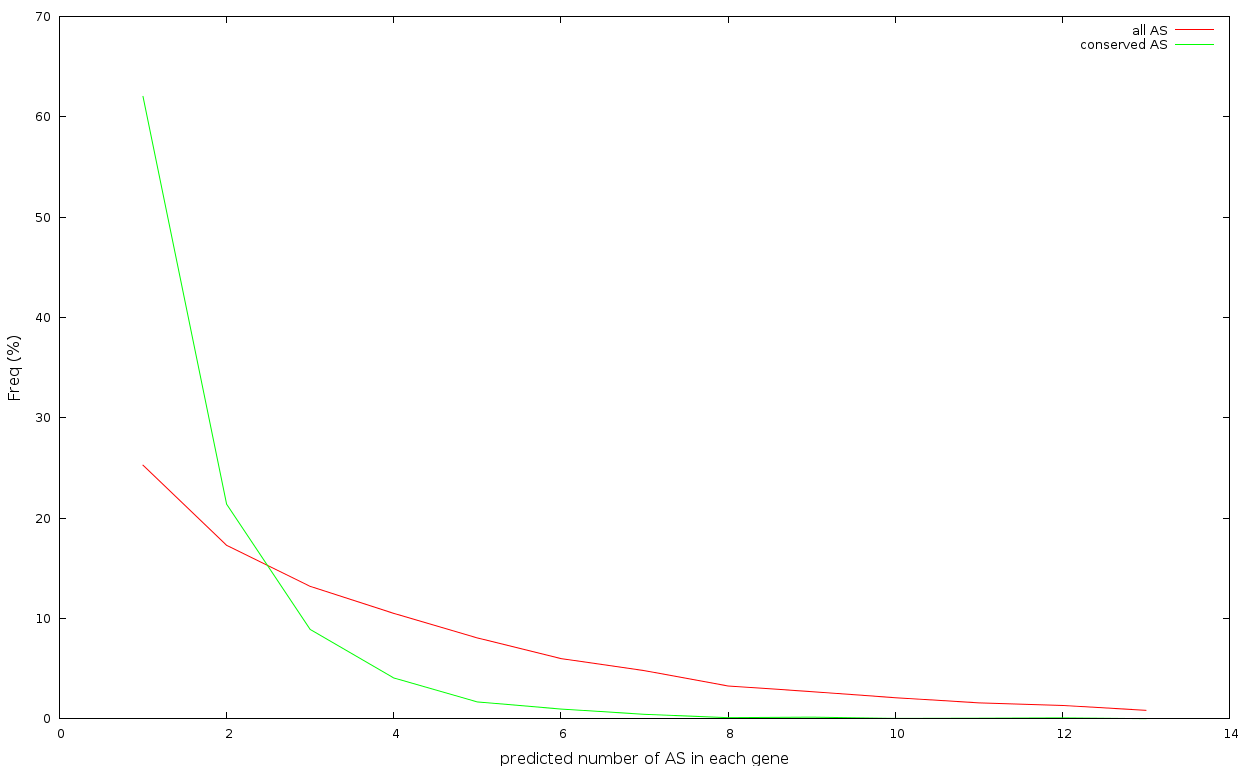


**Figure S3.** Frequency of predicted AS events for a single gene. 11,316 genes are predicted to be AS, 25.27% of which undergo just one AS. The fraction increases up to 62% for genes with conserved AS in more than seven cultivars.


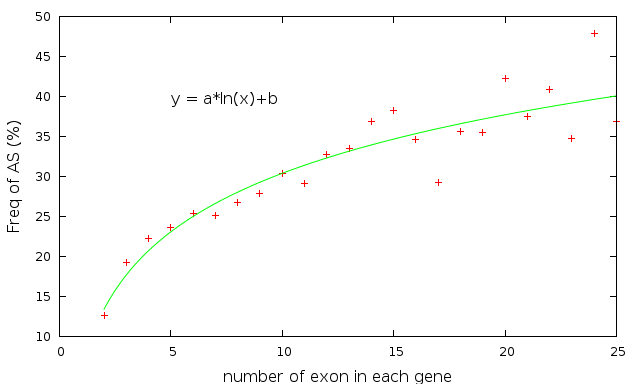


**Figure S4.** Relationship between frequency of AS events and gene exon number. The picture depicts the percentage of genes to be predicted AS among all genes with number of exons given in the x axis, e.g. V2.1 prediction has 5,404 genes with 4 exons, in this class 1,212 genes are predicted to have an AS event. Only exon classes with more than 100 genes are reported. The green line shows the linear regression with parameters a = 10.55 and b = 6.12.


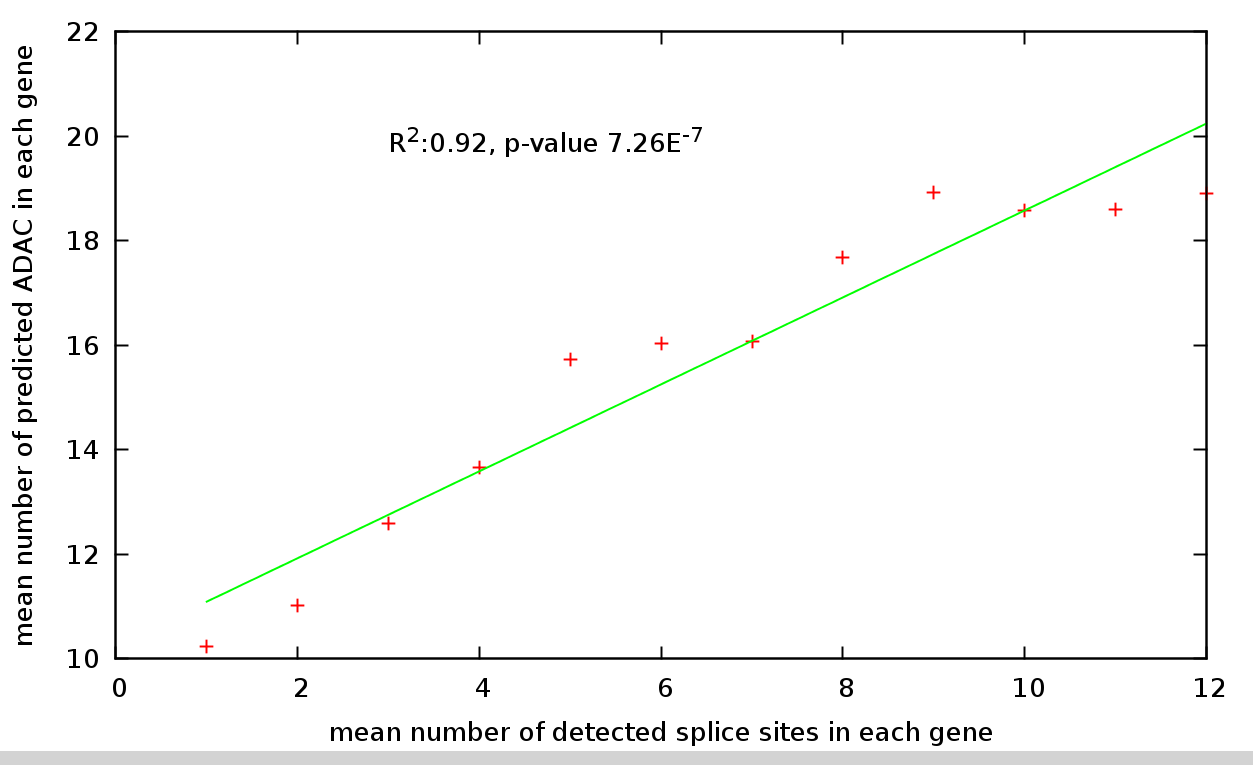


**Figure S5.** Relationship among detected AS events in Pinot noir and predicted AS events in PN40024. For each gene, the number of splice events found is recorded together with the number of predicted AS. Genes with the same number of detected splice events were grouped together and the mean number of AS events for the group plotted. The picture shows only categories with more than 10 genes. The green line represents the linear regression with a p-value of 7.26e-7.


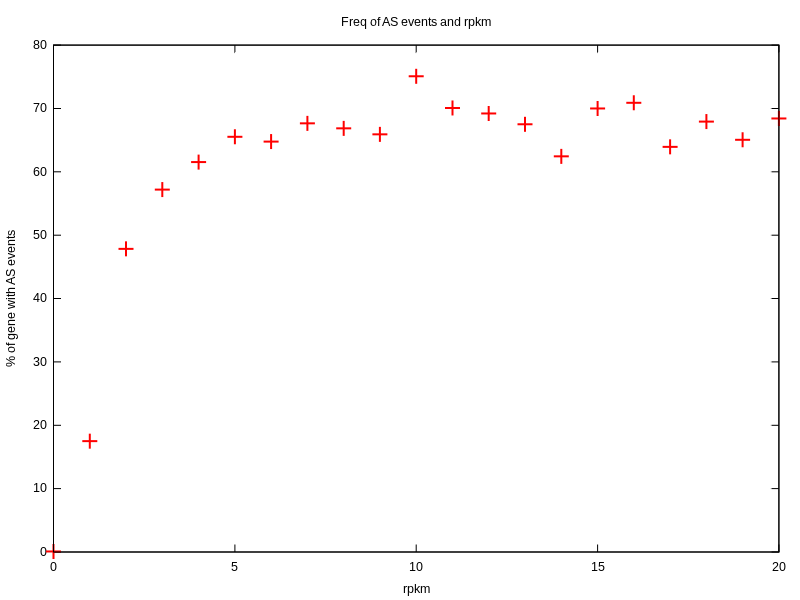


**Figure S6.** Expression level and AS occurrence. Gene expression measure reported as RPKM (reads per kilo base per million) value (x axis) is plotted against the AS events frequency (y axis). Only AS frequencies for RPKM values with more than 100 genes were reported.


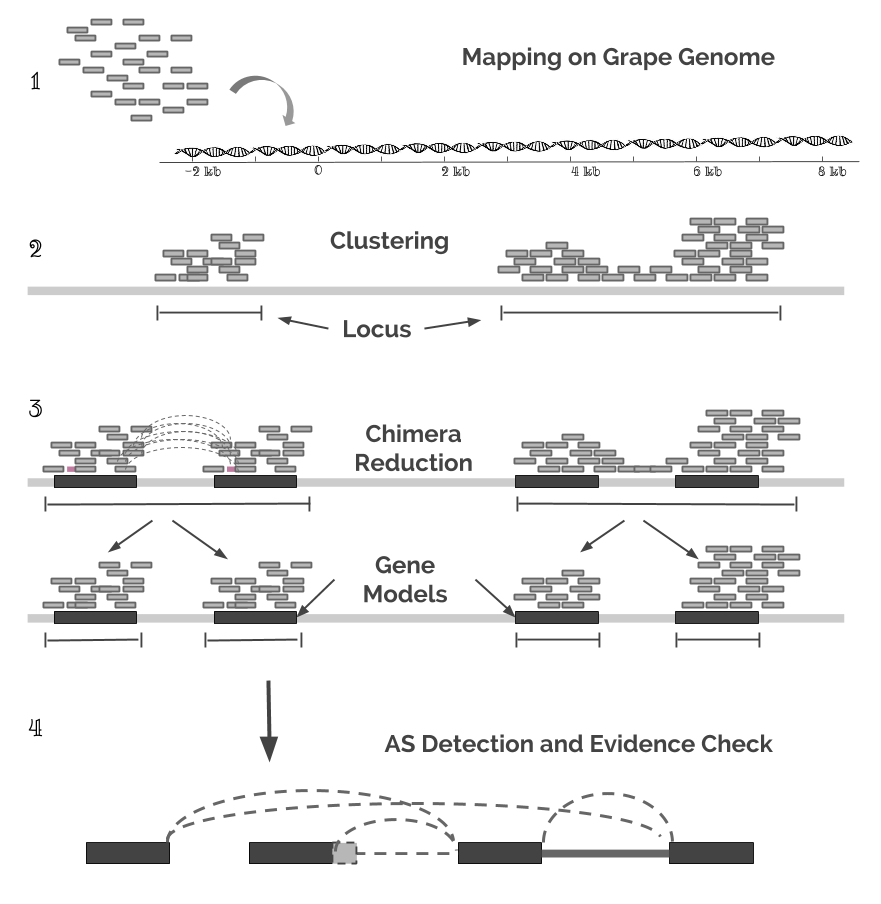


**Figure S7.** findAS workflow. Detection pipeline schematic overview.


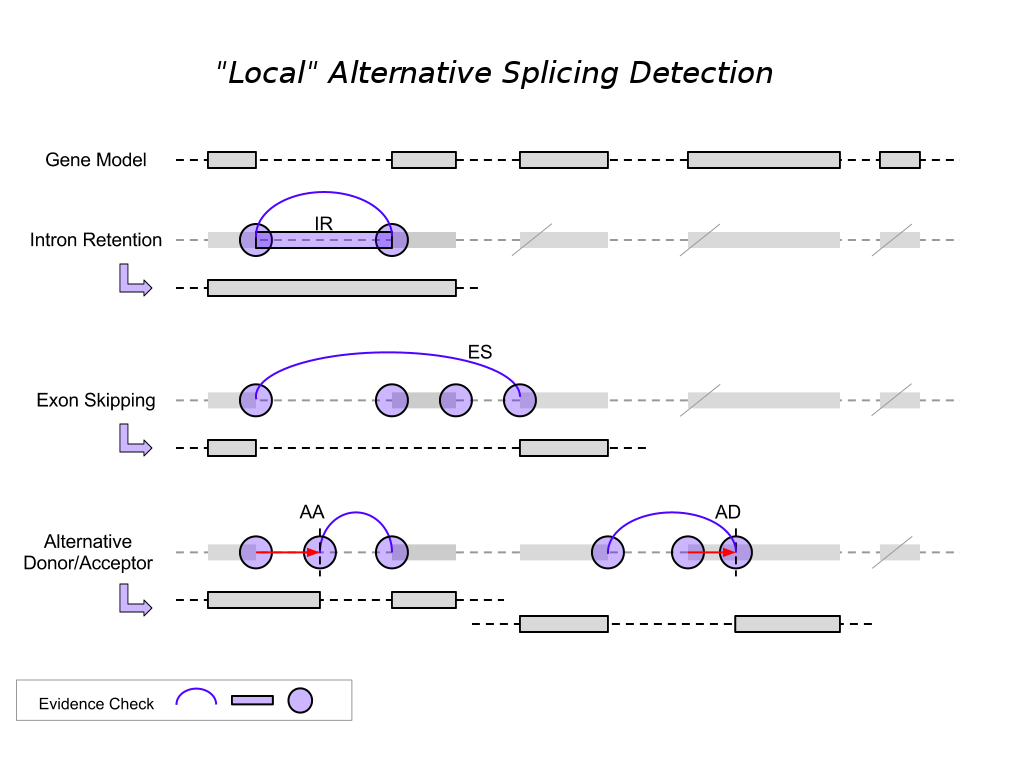


**Figure S8.** Evidence checks. The figure shows positions in which our filter are applied to define an AS event. Intron retention and cryptic introns are kept only if read depth on the purple regions is above the cutoff of 3, with the same cutoff value applied to all other checks. Exon skipping is allowed after evidence checks on the alternative junction and the exon border skipped by the junction. The same checks are then applied to the alternative splice site, alternative junction and constitutive splice site. All the hypotethical AS predicted by FindAS software along with the read counts are available as suppl. mat. file "findas_results.csv".

**Supplementary Figure 9.** Antisense alternative splicing sites. We define a junction as “antisense” when there are evidence of transcription for both strands. For example, if a splice junction on a specific gene prediction is annotates as GT-AG and we found alignment evidence about splice site CT-AC (i.e reverse complement of GT-AG) for the same gene.


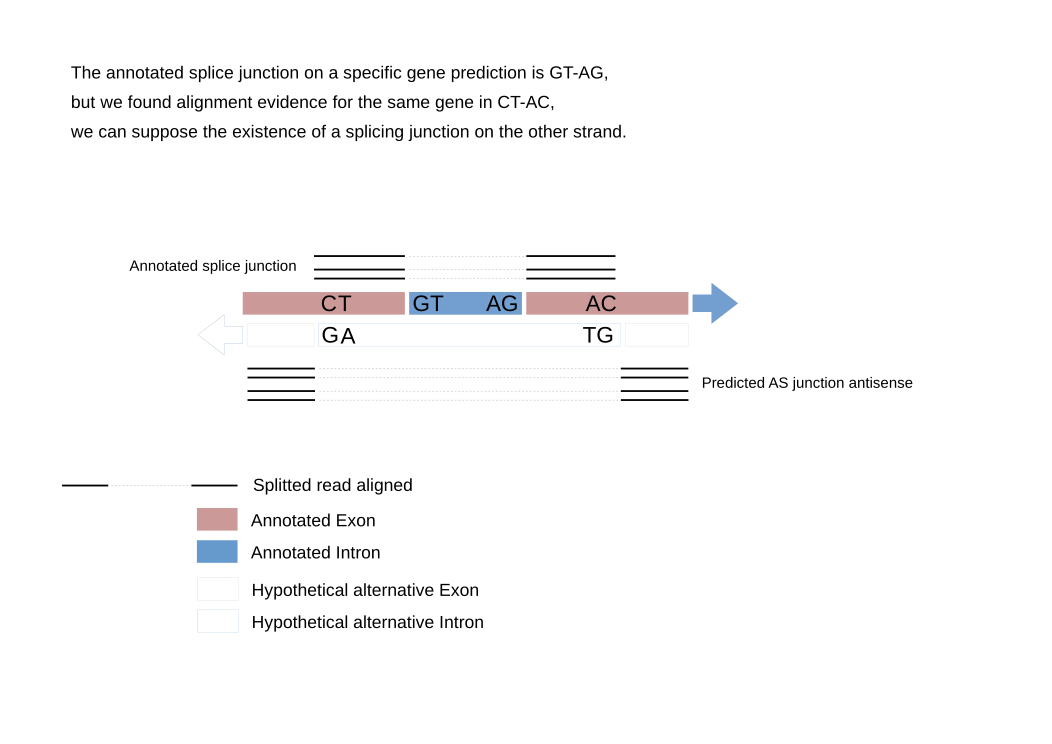


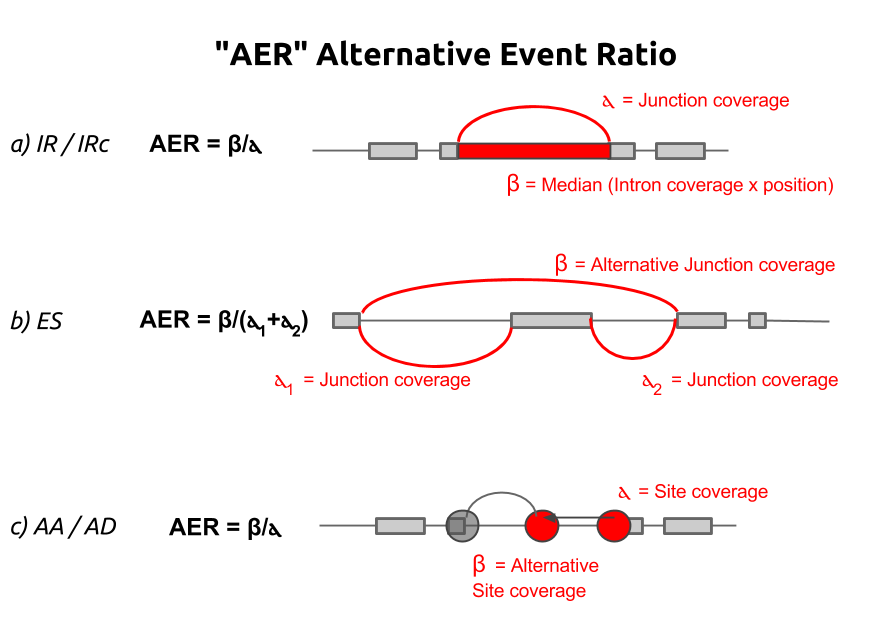


**Figure S10.** Alternative Event Ratio (AER) measures the degree of expression for alternative splicing. a) For intron retention (IR) it is calculated as the median number of reads along the retained intron (α) divided by the number of reads supporting the splice junction (β). b) For exon skipping (ES), AER is calculated as the fraction of alternative junction (β) and the sum of two constitutive junctions (α). c) Alternative Acceptor and Alternative Donor AER is measured as the ratio between the alternative (β) and constitutive splice sites (α).
